# HLA Heterozygote Advantage against HIV-1 Is Driven by Quantitative and Qualitative Differences in HLA Allele-Specific Peptide Presentation

**DOI:** 10.1093/molbev/msz249

**Published:** 2019-10-22

**Authors:** Jatin Arora, Federica Pierini, Paul J McLaren, Mary Carrington, Jacques Fellay, Tobias L Lenz

**Affiliations:** 1 Research Group for Evolutionary Immunogenomics, Max Planck Institute for Evolutionary Biology, Plön, Germany; 2 JC Wilt Infectious Diseases Research Center, National HIV and Retrovirology Laboratory, Public Health Agency of Canada, Winnipeg, MB, Canada; 3 Department of Medical Microbiology and Infectious Diseases, University of Manitoba, Winnipeg, MB, Canada; 4 Basic Science Program, Frederick National Laboratory for Cancer Research, Frederick, MD; 5 Ragon Institute of Massachusetts General Hospital, Massachusetts Institute of Technology and Harvard University, Cambridge, MA; 6 Global Health Institute, School of Life Sciences, École Polytechnique Fédérale de Lausanne, Lausanne, Switzerland; 7 Swiss Institute of Bioinformatics, Lausanne, Switzerland; 8 Precision Medicine Unit, Lausanne University Hospital and University of Lausanne, Lausanne, Switzerland

**Keywords:** major histocompatibility complex, MHC evolution, pathogen-mediated balancing selection, divergent allele advantage, human leukocyte antigen, antigen presentation

## Abstract

Pathogen-mediated balancing selection is regarded as a key driver of host immunogenetic diversity. A hallmark for balancing selection in humans is the heterozygote advantage at genes of the human leukocyte antigen (HLA), resulting in improved HIV-1 control. However, the actual mechanism of the observed heterozygote advantage is still elusive. HLA heterozygotes may present a broader array of antigenic viral peptides to immune cells, possibly resulting in a more efficient cytotoxic T-cell response. Alternatively, heterozygosity may simply increase the chance to carry the most protective HLA alleles, as individual HLA alleles are known to differ substantially in their association with HIV-1 control. Here, we used data from 6,311 HIV-1-infected individuals to explore the relative contribution of quantitative and qualitative aspects of peptide presentation in HLA heterozygote advantage against HIV. Screening the entire HIV-1 proteome, we observed that heterozygous individuals exhibited a broader array of HIV-1 peptides presented by their HLA class I alleles. In addition, viral load was negatively correlated with the breadth of the HIV-1 peptide repertoire bound by an individual’s HLA variants, particularly at *HLA-B*. This suggests that heterozygote advantage at *HLA-B* is at least in part mediated by quantitative peptide presentation. We also observed higher HIV-1 sequence diversity among *HLA-B* heterozygous individuals, suggesting stronger evolutionary pressure from HLA heterozygosity. However, HLA heterozygotes were also more likely to carry certain HLA alleles, including the highly protective HLA-B*57:01 variant, indicating that HLA heterozygote advantage ultimately results from a combination of quantitative and qualitative effects in antigen presentation.

## Introduction

Genes of the major histocompatibility complex (MHC) are a core component of the vertebrate immune system and play a central role in immune-recognition of pathogens. In humans, the classical MHC genes are called human leukocyte antigen (HLA) genes and reside in the MHC region on chromosome 6. MHC genes encode cell-surface molecules that present intra- and extracellular peptides to T cells, which, upon recognizing them as nonself, initiate specific immune responses (Neefjes et al. 2011). Each MHC molecule binds a specific peptide repertoire which is largely defined by the amino acid composition of its peptide-binding groove (Rao et al. 2011; Neefjes and Ovaa 2013). Due to their key role in adaptive immunity and unparalleled allelic diversity within and across vertebrate species, MHC genes have become a paradigm for studying the effect of genetic diversity on immunocompetence and fitness (Nei and Hughes 1991; Sommer 2005; Trowsdale and Knight 2013). The dynamic action of pathogen-mediated balancing selection is widely regarded as a key driver of MHC diversity (Hedrick and Thomson 1983; Apanius et al. 1997; Hughes and Yeager 1998; Solberg et al. 2008; Lenz 2018). Three main mechanisms of pathogen-mediated balancing selection have been proposed: “heterozygote advantage,” “rare-allele advantage,” and “fluctuating selection,” all of which have received empirical support (Sommer 2005; Eizaguirre and Lenz 2010; Spurgin and Richardson 2010; Lenz 2018). It is also largely established that these mechanisms are not mutually exclusive and likely act in parallel to shape the MHC allele pool of a population. However, the relative contribution of each of these mechanisms is still debated and may indeed depend on the specific conditions of a given population or species (Ejsmond and Radwan 2015; Stutz and Bolnick 2017).

First proposed by Doherty and Zinkernagel (1975), the heterozygote advantage hypothesis assumes that heterozygous MHC genotypes confer a higher probability of triggering a specific immune response upon infection. Heterozygosity here (and throughout this article) is defined as carrying two different homologous alleles at a given gene locus that code for different variants of the corresponding molecule. This would result in enhanced pathogen resistance for MHC heterozygous individuals, compared with MHC homozygotes, promoting the persistence of different MHC alleles in the population (Hughes and Yeager 1998; Penn et al. 2002). One possible explanation for heterozygote advantage is the presumed ability of MHC heterozygous individuals to present a broader array of pathogen-derived peptides, thus increasing the probability of inducing a targeted response. This would result in overdominance, where a heterozygote does better than either homozygote. This quantitative explanation for heterozygote advantage has been expanded to the sequence level, triggered by the frequently observed excessive sequence divergence among MHC alleles: the “divergent allele advantage” hypothesis (Potts and Wakeland 1990; Wakeland et al. 1990). It assumes that individuals with more divergent MHC allele combinations (i.e., higher number of amino acid differences between the peptide-binding domains of the two homologous MHC alleles) would express MHC molecules with greater difference in their presented peptide repertoires. This would result in a more diverse array of presented peptides at the cell surface, conferring increased immune-surveillance against pathogens (Lenz 2011; Pierini and Lenz 2018). The observable extent of allele divergence for a given locus can range from 0 (homozygous genotype) to more than 25% of the amino acids in the peptide-binding domain differing between the two alleles, where some alleles that differ only by one or a few amino acids have near-identical binding properties and could be considered homozygous at the functional level. Considering allele divergence instead of simple zygosity thus provides a more nuanced and more functional approach to characterizing MHC variability.

An alternative explanation for heterozygote advantage, based on qualitative differences between MHC alleles, stipulates that heterozygosity increases the probability of carrying specific protective MHC alleles. Such qualitative differences among specific alleles have indeed been observed in a number of species, including humans (Piertney and Oliver 2006; Blackwell et al. 2009; Trowsdale 2011). However, it is unclear whether these qualitative differences among MHC alleles result from unique peptide-binding properties (i.e., the ability to present critical peptides) or whether they are also due to quantitative differences in the size of the allele-specific antigen repertoires (Chappell et al. 2015; Manczinger et al. 2019).

A number of studies across a range of species have provided empirical support for a phenotypic advantage conferred by general MHC heterozygosity (Penn et al. 2002; Takeshima et al. 2008; Evans and Neff 2009; Connor et al. 2010; Niskanen et al. 2014) as well as higher sequence divergence between MHC alleles (Landry et al. 2001; Richman et al. 2001; Neff et al. 2008; Lenz et al. 2009, 2013; Schwensow et al. 2010). Humans have thousands of known alternative HLA alleles (Solberg et al. 2008; Robinson et al. 2015, 2017), yet empirical evidence for pathogen-mediated selection is surprisingly sparse. Owing to the growing number of individuals included in immunogenetic studies and to denser genotyping approaches, multiple significant associations have been identified between infectious or immune phenotypes and variation in the MHC region (Trowsdale 2011; Matzaraki et al. 2017; Meyer et al. 2018). However, most association studies assume simple additive genetic contributions and do not explore the evolutionary implications of their findings, due to a general focus on the underlying biology and disease mechanisms. One of the few exceptions is the seminal study on HIV control by Carrington et al. (1999), which demonstrated a slower progression to HIV-related outcomes (AIDS-defining conditions, CD4^+^ T-cell count <200 and/or death) in HLA heterozygous individuals. Indeed, the role of HLA genes in modulating spontaneous HIV control and progression to AIDS is now well established (Carrington and O’Brien 2003). Recently, the International Collaboration for the Genomics of HIV aggregated an unprecedented data set of HIV-infected individuals to fine-map the association between HLA genetics and HIV control (McLaren et al. 2015). This effort identified *HLA-B* as the major HLA locus in the association with individual variation in HIV-1 set-point viral load (spVL), with three amino acid residues in the antigen-binding groove of *HLA-B* accounting for 11.4% of the variation, followed by two residues of *HLA-A* accounting for an additional 0.9% of variation. These effects appeared to be largely driven by substantial additive associations between various HLA alleles and HIV viral load, but this work nevertheless revealed a small independent protective effect of *HLA-B* heterozygosity. Here, we are therefore revisiting this hallmark example for MHC heterozygote advantage in humans and expand the analysis to all classical HLA class I genes. Furthermore, we characterize the heterozygote advantage at the functional level and explore the relative effect of specific HLA alleles versus a general effect of zygosity on HIV control. We thus take advantage of the well-established association between HLA and HIV control to test whether MHC heterozygote advantage results from quantitative or qualitative differences among MHC alleles. We use genotyping data from 6,311 HIV-1-infected individuals and established antigen-binding prediction algorithms for HLA class I proteins to define the individual repertoires of HIV-1 peptides bound by individual *HLA-A*, *HLA-B*, and *HLA-C* molecule variants. We show that heterozygosity at *HLA-B* and *HLA-C* but not at *HLA-A* is associated with viral control. Although at *HLA-B*, the heterozygote advantage is potentially mediated by quantitative cytotoxic T cell (CTL) response, another mechanism seems to operate at *HLA-C*. Furthermore, we show that specific HLA alleles with very strong effects exceed the general heterozygote advantage against HIV-1.

## Results

The available data comprised HLA genotypes and alleles (imputed at second field resolution, see Materials and Methods) and pretreatment spVL, an established correlate of HIV-1 control and disease progression (Mellors et al. 1996), for 6,311 HIV-infected individuals of European descent. We focused on classical HLA class I genes (**HLA-A**, *HLA-B*, and **HLA-C**), as they are the only genes within the MHC region reported to be independently associated with HIV-1 progression (Carrington et al. 1999; McLaren et al. 2015). A total of 37 *HLA-A*, 69 *HLA-B*, and 27 *HLA-C* alleles were represented in the data set (supplementary tables S1–S3, Supplementary Material online). Using an established algorithm for peptide-binding prediction (NetMHCpan v4.0; Jurtz et al. 2017), we screened all possible 9mer HIV-1 peptides (*N* = 3,252) across the HIV-1 proteome and identified 409, 491, and 223 distinct peptides predicted to be bound by at least one of the represented *HLA-A*, *HLA-B*, and *HLA-C* alleles, respectively (supplementary tables S1–S3, Supplementary Material online).

### HLA Heterozygote Advantage

**Fig. 1.**
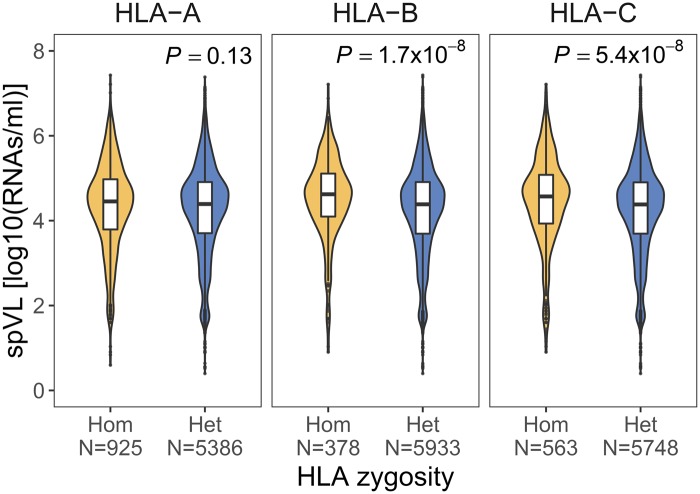
Viral load in HLA homozygotes and heterozygotes. Comparison of the spVL (log10 HIV-1 RNA copies per ml of plasma) between HLA homozygous (Hom) and heterozygous (Het) individuals for the three classical HLA class I loci. *N* indicates the number of individuals. Bonferroni-corrected *P*-value from Wilcoxon rank-sum test is shown.

We first tested whether heterozygosity at any of the classical HLA class I loci was associated with better HIV-1 control (i.e., lower spVL), as reported previously (Carrington et al. 1999). Indeed, we observed a lower level of viral load in both *HLA-B* and *HLA-C* heterozygous individuals, compared with homozygous individuals (Wilcox rank sum test, *P *=* *1.3 × 10^−6^ and 2.8 × 10^−6^ for *HLA-B* and *HLA-C*, respectively, after correcting for multiple testing), whereas heterozygosity at *HLA-A* showed no statistically significant effect on spVL (Wilcox rank sum test, *P *=* *0.11) (fig. 1). Note, however, that even though the associations for *HLA-B* and *HLA-C* were highly significant, the actual effect of heterozygosity on viral load was quite small (effect size = −0.25 and –0.21, respectively). In order to verify that the observed differences in viral load are not caused by the unbalanced sample size for HLA heterozygous and homozygous individuals, we randomly subsampled HLA heterozygous individuals and compared their viral load with an equal number of HLA homozygous individuals 1,000 times. This showed that the lower viral load in HLA heterozygous individuals is not an artifact of unbalanced sample size (supplementary fig. S1, Supplementary Material online).

The MHC region is characterized by strong linkage disequilibrium (LD) between individual HLA genes (Ahmad et al. 2003; Stenzel et al. 2004; Blomhoff et al. 2006). To test whether the observed effects of *HLA-B* and *HLA-C* heterozygosity were independent, we calculated the association of *HLA-B* heterozygosity with spVL among *HLA-C* heterozygotes only (*N* = 5,748), thus controlling for *HLA-C* zygosity. This test demonstrated the effect of *HLA-B* heterozygosity to be independent of *HLA-C* heterozygosity (*P *=* *0.01). The same was true for *HLA-C* zygosity (*P *=* *0.007 within *HLA-B* heterozygotes, *N* = 5,933). The independence of locus-specific effects was also supported when using a linear model that included the heterozygosity at all three classical HLA class I genes as independent factors (supplementary table S4, Supplementary Material online). Moreover, we observed that heterozygosity at two loci led to better HIV-1 control compared with heterozygosity at only one locus (supplementary fig. S2, Supplementary Material online), in line with previous results on survival probability (Carrington et al. 1999). Overall, these results suggest that our data set is appropriate to explore the effect of HLA heterozygote advantage against HIV and its underlying mechanism in more detail.

### Divergent Allele Advantage

**Fig. 2.**
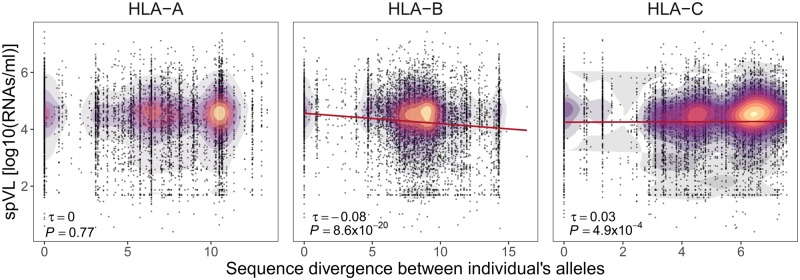
Sequence divergence between individual’s HLA alleles and viral load. Correlation between spVL (log10 HIV-1 RNA copies per ml of plasma) and sequence divergence between individual’s *HLA-A*, *HLA-B*, and *HLA-C* alleles is shown (including homo- and heterozygotes; *N* = 6,311). The color indicates the density of individuals. Kendall’s estimate of correlation τ and Bonferroni-corrected *P*-value are shown.

We next sought to test the hypothesis of divergent allele advantage by evaluating the relation between sequence divergence of an individual’s allele pair and viral load. In our data set, the number of amino acids that differed between the antigen-binding domains of an individual’s two homologous alleles at a given HLA locus ranged from 0 (homozygous genotypes, i.e., two identical alleles) to 28 (15.5%), 31 (17.1%), and 19 (10.5%) amino acids at *HLA-A*, *HLA-B*, and *HLA-C*, respectively. In order to capture differences in the physiochemical properties among amino acids, we calculated sequence divergence using the Grantham distance score. The Grantham score is based on differences in physiochemical properties as well as in the spherical volume among amino acids, and in an earlier study proved to be the most suitable proxy for HLA functional divergence among several different amino acid similarity scores (Pierini and Lenz 2018). First, we saw that, although zygosity correlated strongly between the *HLA-B* locus and the other two HLA class I loci, *HLA-C* and *HLA-A* (τ = 0.55, *P *<* *1 × 10^−64^ and τ = 0.16, *P *=* *1.1 × 10^−37^, respectively), the sequence divergence between an individual’s *HLA-B* alleles was only weakly correlated with the divergence between *HLA-C* (τ = 0.09, *P *<* *0.0001) and *HLA-A* alleles (τ = 0.03, *P *=* *0.002) (supplementary fig. S3, Supplementary Material online). This might suggest both different mutational mechanisms in generating sequence divergence and independent selection processes maintaining sequence divergence at these loci, in line with selection usually targeting very specific residues involved in peptide binding (Reche and Reinherz 2003). Following the predictions of the divergent allele advantage hypothesis, *HLA-B*, the locus with by far the strongest association to HIV control, showed a negative correlation between pairwise allele divergence and spVL across individuals (τ = −0.08, *P *=* *8.6 × 10^−20^) (fig. 2). Although we found no such correlation for *HLA-A* (*P *=* *0.77), *HLA-C* surprisingly showed a positive association between allele divergence and viral load (τ = 0.03, *P *=* *4.9 × 10^−4^). We observed similar correlations after excluding homozygous individuals (supplementary fig. S4, Supplementary Material online). In addition, the correlation between viral load and the interallele divergence at classical HLA class I genes was supported by their respective effect size calculated using a linear model in which the sequence divergence between individuals’ *HLA-A*, *HLA-B*, and *HLA-C* together were taken as predictor variables (supplementary table S5, Supplementary Material online). The coefficients of this linear model also allowed us to estimate the actual quantitative effect of HLA allele divergence on viral load. Using data from Pierini and Lenz (2018), we calculated that one unit of Grantham divergence on average corresponds to about 2.2 amino acids difference between two HLA class I alleles. With a linear coefficient of −0.04 for *HLA-B* divergence (supplementary table S5, Supplementary Material online), this means that one amino acid difference between an individual’s two *HLA-B* alleles corresponds to a decrease in log HIV-1 RNA copies per ml blood of about −0.006. It is difficult to judge whether such a decrease in viral RNA copies provides a substantial benefit for an infected individual, and whether such differences could contribute to selection on HLA genes. However, over evolutionary time-scales, even small selection coefficients can have significant effects.

Noteworthy with regard to the positive correlation for *HLA-C*, alleles of the HLA-C*07 subgroup are known to confer susceptibility to HIV because of their low expression on the cell surface (Kulkarni et al. 2011), an effect we can also observe in our data (supplementary fig. S5*A*, Supplementary Material online). Coincidentally, these alleles are also highly divergent from the rest of the *HLA-C* alleles (Robinson et al. 2017). Indeed, individuals carrying an allele of the HLA-C*07 subgroup have significantly more divergent *HLA-C* genotypes than individuals that do not carry such alleles (supplementary fig. S5*B*, Supplementary Material online). The higher spVL of highly divergent *HLA-C* genotypes might thus be due to low cell-surface expression of *HLA-C*, a mechanism unrelated to the diversity of presented HIV peptides. Notably, even though *HLA-B* and *HLA-C* loci are in strong LD, the pairwise Grantham distance between individual’s *HLA-B* alleles was significantly higher than between *HLA-C* alleles (*P *<* *0.001) (supplementary fig. S6, Supplementary Material online), supporting the notion that *HLA-C* is not evolving under the same selective constraints as *HLA-B*. Furthermore, *HLA-C* generally does not seem to be under selection for contributing to diverse immunological surveillance (Buhler et al. 2016; Robinson et al. 2017). It is thus possible that this positive correlation is not due to quantitative differences in antigen presentation among *HLA-C* alleles, but due to other mechanisms, for instance variation in cell-surface expression or the known interactions between *HLA-C* and Killer-cell immunoglobulin-like receptor (KIR) (Parham et al. 2012).

### Functional Consequence of Heterozygosity and Allele Divergence

**Fig. 3.**
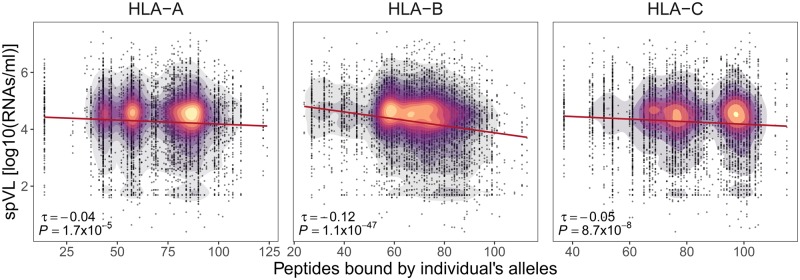
HLA-bound peptides and viral load (spVL). Correlation between individual’s spVL (log10 HIV-1 RNA copies/ml of plasma) and the breadth of HIV-1 peptides predicted to be bound by the individual's *HLA-A*, *HLA-B*, and *HLA-C* alleles is shown (including homo- and heterozygotes; *N* = 6,311). The color indicates the density of individuals. Kendall’s estimate of correlation τ and Bonferroni-corrected *P*-value are shown.

Having observed the effects of both heterozygosity and allele divergence in our data, we then asked whether these measures of genetic variability would indeed allow for the presentation of a broader array of HLA-bound peptides, as hypothesized by the quantitative explanation for MHC heterozygote advantage. Using computational peptide-binding prediction, we found that heterozygosity on average resulted in a broader array of bound peptides for all three classical HLA loci (supplementary fig. S7, Supplementary Material online). Furthermore, the number of peptides bound by a pair of HLA alleles was positively correlated with the sequence divergence between the alleles at all three HLA loci (supplementary fig. S8, Supplementary Material online). This association between sequence divergence at the HLA loci and predicted functional divergence among HLA molecule variants has been reported before (Lenz 2011; Pierini and Lenz 2018). However, our present HIV data set allowed us to evaluate this association using an empirical disease phenotype. We thus tested whether the ability to bind more HIV-1 peptides was associated with HIV control (i.e., spVL). Indeed, the individual-specific number of HIV-1 peptides predicted to be bound by the individual’s *HLA-B* molecules was negatively associated with viral load (Kendall correlation, τ = −0.12, *P *=* *1.1 × 10^−47^) (fig. 3). This was also true for *HLA-A* and *HLA-C*, but correlation coefficients were smaller (Kendall correlation, *HLA-A*: τ = −0.04, *P *=* *1.7 × 10^−5^; *HLA-C*: τ = −0.05, *P *=* *8.7 × 10^−8^). The correlation between viral load and predicted number of HIV-1 peptides presented by classical HLA class I molecules was supported by their respective effect size calculated using a linear model in which the number of HIV-1 peptides bound by individuals’ *HLA-A*, *HLA-B*, and *HLA-C* together were taken as predictor variables (supplementary table S6, Supplementary Material online). The linear ß-coefficient for *HLA-B* of −0.012 means that each additional HIV-1 peptide bound by *HLA-B* leads to a decrease in log HIV-1 RNA copies per ml blood of about –0.007. Interestingly, the association between viral load and the breadth of individual-specific predicted *HLA-B* bound peptides was stronger (τ = −0.12) than the association between viral load and allele divergence (τ = −0.08), suggesting that allele divergence is a useful but imperfect proxy for functional divergence among *HLA-B* alleles, at least in the case of a single pathogen with a limited peptide repertoire, such as HIV-1.

In order to assure that these results are not biased by the selected affinity threshold for binding between HLA and HIV-1 peptides, we evaluated the association of HLA-bound HIV-1 peptides also with a more stringent and a more relaxed affinity threshold binding. Here, we observed an equivalent association between the viral load and the number of HLA-bound HIV-1 peptides (supplementary fig. S9, Supplementary Material online). Moreover, given that some HLA alleles can bind non-9mer peptides, we extended this analysis to 8mer and 10mer HIV-1 peptides. We observed similar negative associations between viral load and predicted HLA-bound 10mer peptides (supplementary fig. S10, Supplementary Material online), but not for 8mer peptides. This is likely due to the much smaller number of bound 8mer peptides per HLA allele (median 2 ± 3 SD) compared with 9mer (41 ± 10) or 10mer (20 ± 10) peptides.

### HLA Diversity and Within-Host Evolution of HIV

**Fig. 4.**
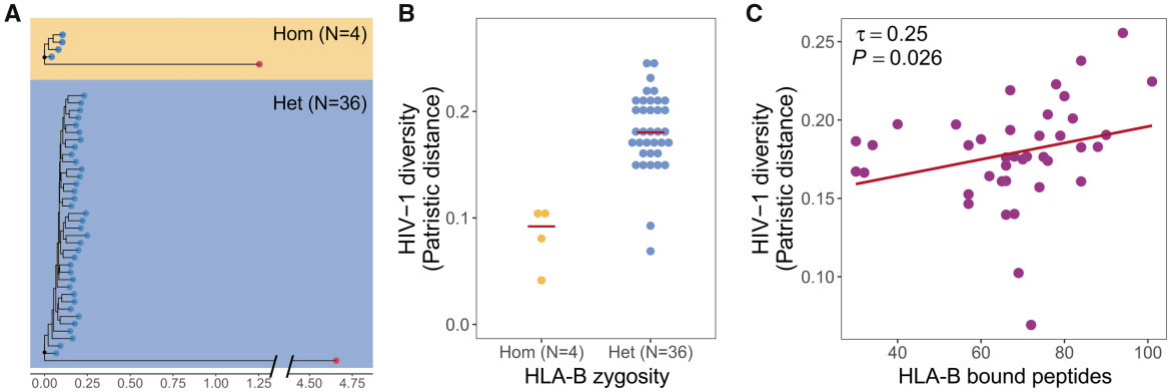
Evolution of HIV sequence diversity in response to *HLA-B* diversity. (*A*) Rooted phylogenetic trees of autologous virus sequences (blue dots) from *HLA-B* homozygous (*N* = 4) and heterozygous (*N* = 36) individuals. We used HIV-2 (red dot) as outgroup to identify the root during tree construction. (*B*) Virus sequences exhibit higher sequence diversity (measured root-to-tip patristic distance, i.e., sum of branch lengths) among *HLA-B* heterozygous individuals (blue dots) compared with homozygotes (yellow dots). *N* indicates the number of individuals. (*C*) The number of predicted *HLA-B* bound peptides correlates positively with the diversity of HIV sequences among individuals (*N* = 40, correlation coefficient τ and *P*-value from Kendall rank correlation are shown).

Presentation of pathogen-derived peptides by HLA molecules is thought to increase the likelihood and efficiency of a pathogen-specific immune response. Consequently, such HLA restriction is a potential factor that might influence the evolutionary landscape of pathogens. Specifically for HIV-1, the virus has been shown to acquire mutations within HLA-bound peptides that can help it to escape immune recognition (Leslie et al. 2004; Bronke et al. 2013; Arora et al. 2019). In this context, following the predictions of the quantitative heterozygote advantage hypothesis, heterozygous HLA genotypes should exert a broader selective pressure on the virus, leading to a larger number of escape mutants. Taking advantage of our unique data set, which also comprised a limited set of autologous HIV-1 sequences from a subset of individuals (*N* = 40, one HIV-1 sequence per individual; see Materials and Methods), we performed a phylogenetic comparison of these autologous HIV-1 sequences to test whether HLA heterozygosity leads to more pronounced sequence evolution, possibly as an adaptation because of the broader HLA restriction in HLA heterozygous individuals. For this analysis we focused on *HLA-B*, the locus with strongest association with HIV control, and observed that autologous virus sequences in *HLA-B* heterozygous individuals (*N* = 36) show indeed higher sequence diversity than the ones in homozygous individuals (*N* = 4; HIV diversity measured as root-to-tip distance, fig. 4*A* and *B*). In order to account for the unbalanced sample size, we permuted the individuals across zygosity groups for 10,000 times and each time recalculated the average root-to-tip distances. This analysis showed that the observed difference was unlikely to be due to chance (one-tailed *P *=* *0.027) (supplementary fig. S11, Supplementary Material online). However, the bias in the sample sizes from the small number of homozygous subjects remains a problem for this analysis. We therefore tested the above prediction also in a different approach that is not hampered by biased sample sizes. For this, we tested the effect of the overall breadth of HLA-presented peptides on virus evolution. Again, HLA genotypes that allow presentation of a broader set of HIV-1 peptides should lead to more pronounced evolution in the virus. Accordingly, we observed a positive correlation between the total number of predicted HLA-bound peptides and the diversity of HIV sequences among individuals for which autologous HIV sequence data were available (*N* = 40) (fig. 4*C*). In a previous analysis of these data, we already identified six mutations in HIV-1 that were predicted to evade peptide presentation by the variant HLA-B*57:01 (Arora et al. 2019), likely contributing to the elevated sequence diversity in HLA heterozygotes observed here. Nonetheless, more autologous HIV-1 sequences, particularly from HLA homozygous individuals, will help to corroborate these findings.

### Allele-Specific Effects versus General Heterozygote Advantage

**Fig. 5.**
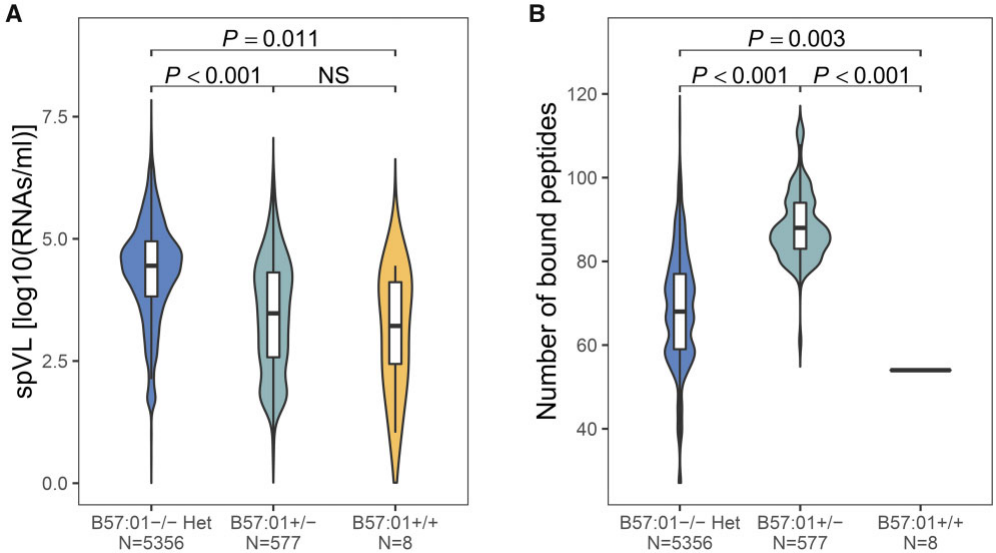
Heterozygote advantage versus allele-specific effect for HLA-B*57:01. Variation in (*A*) set point viral load (spVL, log10 HIV-1 RNA copies/ml of plasma) and (*B*) the number of HIV-1 peptides bound by *HLA-B* in HLA heterozygous individuals not carrying HLA-B*57:01 (B57:01−/−), individuals carrying one copy of HLA-B*57:01 (B57:01+/−), and individuals homozygous for HLA-B*57:01 (B57:01+/+). *N* indicates the number of individuals. Bonferroni-corrected *P*-values from Wilcoxon rank sum test are shown.

HLA alleles are known to show differential association with HIV-1 control ranging from risk to protection (McLaren et al. 2015). We therefore tested the alternative hypothesis for heterozygote advantage, which suggests that heterozygosity might simply increase the chances of carrying a particular HLA allele that binds immunogenic peptides and through that provides better viral control. Of seven *HLA-B* alleles significantly associated with HIV control, four (including B*57:01) were indeed enriched in the heterozygous state (supplementary fig. S12, Supplementary Material online), making this hypothesis a viable explanation. The nonsignificant enrichment for the other three alleles could be due to their low frequency in our data set. We then asked what property made individual alleles more protective. Following the same intuition as for the quantitative heterozygote advantage above, they could confer protection simply by presenting more peptides to T cells compared with other alleles. Alternatively, they could confer a qualitative advantage by presenting very specific peptides that are particularly difficult (i.e., costly in terms of fitness) for the virus to mutate. We tested this point by focusing on HLA-B*57:01, the allele known to confer the strongest resistance against disease progression (Migueles et al. 2000; Bailey et al. 2006; Pohlmeyer et al. 2013; McLaren et al. 2015). We found that heterozygous individuals with the allele B*57:01 exhibited a lower viral load than heterozygotes without this allele (carrying any two *HLA-B* alleles except B*57:01) (Wilcoxon rank sum test, *P *<* *0.001; fig. 5*A*). However, heterozygotes with B*57:01 are also predicted to bind a greater breadth of peptides compared with heterozygous individuals without B*57:01 (Wilcoxon rank sum test, *P *<* *0.001; fig. 5*B*), making it difficult to discern quantitative and qualitative effects of B*57:01. Yet, homozygotes with the allele B*57:01 alone were predicted to bind fewer HIV-1 peptides (54 peptides) than heterozygotes without this allele (binding a median of 68 peptides; Wilcoxon rank sum test, *P *=* *0.003; fig. 5*B*). This allowed us to evaluate the qualitative effect of B*57:01 in binding specific HIV peptides on viral load while excluding any quantitative advantage of binding more HIV peptides. We found that individuals homozygous for B*57:01 still exhibited a lower viral load than B*57:01−/− heterozygotes (Wilcoxon rank sum test, *P *=* *0.011) (fig. 5*A*), despite the fewer peptides they can present, suggesting that binding of specific HIV-1 peptides provides a qualitative advantage to B*57:01. Nevertheless, B*57:01 is also predicted to bind a higher than average number of HIV-1 peptides among all tested *HLA-B* alleles (Arora et al. 2019), maintaining the possibility that both qualitative and quantitative aspects of peptide binding are contributing to the protective effect of this allele.

Having established that the effect of individual HLA alleles can significantly exceed the effect of general zygosity, we next aimed to explore more generally the relative role of additive effects of *HLA-B* alleles versus *HLA-B* heterozygosity. An independent advantageous effect of *HLA-B* heterozygosity had already been shown in a previous fine-mapping study (McLaren et al. 2015). Here, we therefore compared the observed association of HLA heterozygosity with spVL to the effects of HLA sequence divergence and the number of bound HIV-1 peptides after accounting for the additive effect of individual *HLA-B* alleles. Using a regression model that included allele-specific effects in an additive manner, we observed qualitatively equivalent effects of HLA heterozygosity, sequence divergence, and the number of predicted HLA-bound peptides on viral load as in the absence of allele-specific effects, with a variation in viral load associated with these compound parameters ranging from 0.06% to 0.09% (table 1). Noteworthy, the beneficial effect of heterozygosity remains significant overall, even though we found above that there is no significant difference in viral load between homozygotes and heterozygotes among carriers of the allele HLA-B*57:01, likely because of the dominant protective effect of this particular allele. This suggests in turn that heterozygosity is even more beneficial among carriers of other alleles than shown by these numbers. However, it is also apparent that the independent effect of these compound parameters is substantially lower than the combined additive effects of all individual *HLA-B* alleles, which account for 11.4% of the variation in viral load (McLaren et al. 2015). Interestingly, after including allele-specific additive effects, which account for the allele-specific HIV-1 peptide repertoire size, the effect of *HLA-B* sequence divergence and predicted combined number of bound HIV-1 peptides becomes very similar. This suggests that allele divergence (here measured as Grantham distance) is a useful proxy for the overlap in binding properties, that is, the redundancy in presented peptides between two HLA alleles.

**Table 1.** Variation in HIV-1 set point viral load (spVL) associated with different measures of *HLA-B* diversity before and after accounting for allele-specific additive effects.

	Associated Variation in spVL in % (*P*-value)	
*HLA-B*	Without Allele-Specific Effects	With Allele-Specific Effects
Heterozygosity	0.3 (6.6 × 10^−6^)	0.06 (0.016)
Sequence divergence	1.3 (1.0 × 10^−20^)	0.09 (0.005)
Bound peptides	3.3 (2.6 × 10^−50^)	0.09 (0.006)

note.—Effect of *HLA-B* heterozygosity, sequence divergence between individual *HLA-B* alleles, and breadth of predicted *HLA-B* bound HIV-1 peptides is shown. *P*-values from linear regression models are given in parentheses (*N* = 6,311).

## Discussion

Of all three classical HLA class I genes tested here, heterozygosity at *HLA-B* and *HLA-C* was independently associated with viral control. The absence of a significant association with *HLA-A* heterozygosity that contrasts with a previously observed association between *HLA-A* heterozygosity and disease progression (Carrington et al. 1999) might either be owing to its genetic linkage (LD) with neighboring *HLA-B* and/or *HLA-C* loci (Ahmad et al. 2003; Stenzel et al. 2004; Blomhoff et al. 2006) or the different disease parameters used (progression over time vs. spVL). However, the absence of a statistically significant association between sequence variation at *HLA-A* and HIV-1 viral load might also suggest that *HLA-A* is not a prime target of pathogen-mediated selection, which was already proposed earlier and is supported by the conserved profile of *HLA-A* residues involved in peptide binding (dos Santos Francisco et al. 2015). We also recapitulated the observation that higher sequence divergence between an HLA allele pair generally could lead to a larger number of bound peptides, but here focusing only on HIV peptides (Lenz 2011; Pierini and Lenz 2018). The weaker association of sequence divergence with HIV-1 viral load suggests that the number of bound peptides, where available, could be a better proxy for immunocompetence when focusing on a specific pathogen, particularly if it has a small proteome as was the case here.

The negative correlation between the number of *HLA-B*-bound peptides and viral load among individuals suggests that *HLA-B* heterozygote advantage is significantly mediated via quantitative CTL response to a broad set of HLA-presented HIV-1 peptides, though empirical validation would substantiate the finding. Interestingly, a relatively weak negative correlation between *HLA-C* bound peptides and viral load suggests that an effector mechanism other than CTL-mediated quantitative immune response might be responsible for *HLA-C* heterozygote advantage. This suggestion gains additional support from the fine-mapping study by McLaren et al. (2015), where unlike for *HLA-B*, there were no HIV-associated amino acid residues found for *HLA-C*. Moreover, with only about half the peptide-repertoire size of *HLA-C* alleles, relative to *HLA-A* and *HLA-B*, *HLA-C* might be evolving not to interact with the vast diversity of CTLs, but other relatively less diverse cell types. One such cell type could be natural killer cells which express KIRs on their cell surface (Parham 2005). *HLA-C* molecules are thought to be a potent ligand of KIRs (Colonna et al. 1993; Parham 2005; Hilton et al. 2015), and specific interactions between *HLA-C* molecules and KIRs have been associated with multiple diseases, including HIV infection (Rajagopalan and Long 2005; Zipeto and Beretta 2012; Körner et al. 2017).

Pereyra et al. (2014) have shown that CD8^+^ T-cell targeting of specific HLA-presented peptides could confer viral control (Pereyra et al. 2014). A broader array of HLA-bound peptides in heterozygous individuals might increase the possibility that such peptides are presented on the cell surface. However, the particular case of the B*57:01 allele conferring superior viral control compared with general *HLA-B* heterozygote advantage suggests that HLA allele-specific effects might arise from binding specific immunodominant peptides. Nevertheless, as B*57:01 also bound the largest number of peptides among all *HLA-B* alleles, the quantitative advantage of binding a large number of peptides might contribute to its strong protective effect in HIV-1 control.

Together, these results suggest that HLA heterozygosity in an individual might confer advantage in multiple, possibly additive ways. One is the quantitative advantage through presentation of a larger number of viral peptides, which might generate a broader immune response. This appears to exert stronger evolutionary pressure on the virus to evolve, as shown by elevated sequence diversity of the virus among HLA heterozygous individuals, possibly resulting in replicative fitness cost. In addition, HLA heterozygosity might provide an advantage by making it more likely to carry certain protective HLA alleles that can present immunodominant peptides to T cells and thus lead to disease control.

In conclusion, our findings shed light on the functional basis of the protective association between HLA heterozygosity and HIV control. Interestingly, heterozygote advantage is generally thought to be more important in a multiparasite context, with HLA heterozygosity assumed to enable hosts to recognize and fight more different parasites (Penn et al. 2002). It is certainly conceivable that in such a multiparasite context, quantitative aspects of antigen presentation become more important than qualitative aspects due to the sheer number of peptides involved. Nevertheless, our study demonstrates and characterizes HLA gene-specific heterozygote advantage even against a single pathogen. The findings disentangle the role of quantitative and qualitative features of the HLA’s peptide repertoire in mediating the immune response and suggest that even a single pathogen can lead to selection for both HLA heterozygosity (including excessive allele divergence) and specific HLA alleles. Moreover, they lend support to HIV vaccine programs aiming to impart antiviral immunity using a broad, yet specific array of HIV peptides.

## Materials and Methods

### Samples and Genotype Data

We used HLA genotyping and clinical data from 6,311 chronically HIV-1-infected individuals that were previously analyzed. HLA genotype imputation and quality control are described in detail in McLaren et al. (2015). Briefly, genome-wide single nucleotide polymorphism (SNP) genotype data were collected from eight genome-wide association studies mentioned in supplementary table S7, Supplementary Material online. Originally, the data were sequenced in 21 cohorts across Australia, Europe, and the United States participating in the International Collaboration for the Genomics of HIV, through which the data can be accessed. SNP genotypes absent in original genotyping platforms were imputed (McLaren et al. 2015). Imputed SNPs with low imputation quality (*R*^2^ score < 0.3) or minor allele frequency of <0.5% were discarded. HLA alleles (at second-field resolution) for classical class I loci (**HLA-A**, *HLA-B*, **HLA-C**) were imputed as best-guess genotypes from genome-wide genotype data using the SNP2HLA method (Jia et al. 2013) and a reference panel consisting of 5,225 individuals of European ancestry from the Type-1 Diabetes Genetics Consortium (T1DGC) (Rich et al. 2006). Following an established HLA imputation procedure, individuals whose alleles scored a robust *R*^2^ (imputation quality score) >0.8 were retained, yielding a data set with 6,311 individuals with a total of 37 *HLA-A*, 69 *HLA-B*, and 27 *HLA-C* alleles. Pretreatment HIV-1 spVL (log10 HIV-1 RNA copies/ml of plasma) was used as a quantitative disease phenotype (McLaren et al. 2015).

### HLA Binding Affinity for HIV-1 Peptides

Following Arora et al. (2019), we used the reference proteome of HIV-1 M group subtype B (NCBI accession number NC_001802.1) that comprised ten proteins. The *Gag–Pol* protein is a precursor protein resulting from a −1 ribosomal frameshifting event in upstream *Gag* (Jacks et al. 1988), and then cleaved by the virus-encoded protease to produce the mature *Pol* protein. In order to avoid redundancy with the separate *Gag* protein in our analysis, we manually trimmed the *Gag–Pol* protein sequence to *Pol*. HLA class I molecules preferentially bind and present 9mer peptides (Falk et al. 1991; York et al. 2002). We used a computational method called NetMHCpan v4.0 (Jurtz et al. 2017) to predict the binding affinity of all possible 9mer peptides derived from the entire HIV-1 proteome to individual HLA class I alleles represented in our data set. The method reports the rank of predicted binding affinity of HLA-peptide complexes against predicted affinity of random natural peptides. HLA-peptide complexes with predicted binding affinity rank <0.5 were retained (corresponding to “strongly bound” peptides) (Jurtz et al. 2017). The breadth of peptides bound by an individual’s HLA allele pair was taken as the total number of unique peptides predicted to be bound by both alleles.

### Sequence Divergence between Alleles of HLA Genotype per Individual

Sequence divergence between alleles was computed for all HLA allele pairs (genotypes) of *HLA-A*, *HLA-B*, and *HLA-C* loci. Protein sequences of HLA alleles were taken from IMGT/HLA database (Robinson et al. 2015). Exons 2 and 3, which encode the variable region in the peptide-binding groove of HLA class I molecules, were obtained following the exon annotation reported in Ensemble database (Aken et al. 2016). The alignment of amino acid sequences was performed using MUSCLE (Edgar 2004). The genetic distances between aligned allele pairs were calculated based on the Grantham distance matrix (Grantham 1974) using a custom Perl script freely available online (Pierini and Lenz 2018). The nonparametric Kendall correlation was used to test for the associations of the sequence divergence between individual’s HLA alleles with 1) spVL and 2) the combined number of predicted HLA-bound peptides. All *P*-values were adjusted for multiple testing across the number of loci tested.

### Phylogenetic Comparison of Autologous Virus Sequences

Autologous amino acid sequences of six HIV-1 proteins, namely *Gag*, *Pol*, *Vif*, *Vpr*, *Vpu*, and *Nef*, were obtained from bulk sequencing of viral RNA as described in Bartha et al. (2013) and available for 65 individuals (one consensus sequence per individual). Due to fragmented coverage of sequences, we concatenated these six proteins in order to obtain better resolution and statistical power and aligned them using MAFFT v7 with default parameters (Katoh et al. 2002). However, the viral sequences were too fragmented (missing sequence information), and this introduced several gaps in their alignment. Therefore, we optimized the alignment for maximum gap-free area by selecting an optimal subset of sequences using MaxAlign-1.1 (Gouveia-Oliveira et al. 2007), which resulted in sequence data from 4 *HLA-B* homozygous and 36 heterozygous individuals (one autologous sequence each). Maximum likelihood trees were made using PhyML-3.1 (Guindon and Gascuel 2003) with default parameters. The tip-to-tip distances were extracted using Ape-3.5 package (Paradis et al. 2004) in R 3.5.1. Using HIV-2 as an outgroup, the root-to-tip distance was used as a proxy for evolutionary diversity among host-specific HIV-1 clones. Difference in the mean root-to-tip distance in each group of individuals was taken as the observed difference in diversity. We obtained statistical significance of the observed difference by permuting the individuals across the groups and repeating the above procedure 10,000 times. *P*-value was taken as the fraction of permutations where the difference in mean divergence was equal to or more than the observed difference.

### Association with Viral Load While Controlling for Allele-Specific Effects

The association of a variable with spVL was calculated using a linear regression model following McLaren et al. (2015). Variation in spVL attributable to a given variable (heterozygosity, sequence divergence, or the breadth of predicted HLA-bound peptides) while controlling for allele-specific effects was taken as the difference between adjusted-*R*^2^ values of the model with variable, alleles and covariates (eq. 1) and the model with alleles and covariates only (eq. 2). We did the analysis for each classical HLA class I locus separately. Models contained all imputed alleles (*N* = 69 for *HLA-B*, *N* = 37 for *HLA-A*, *N* = 27 for *HLA-C*), the first five principle components of SNP variation, and the cohort identity (all adopted from McLaren et al. [2015]) as the covariates. The significance of the variable’s association with viral load was calculated by comparing these two models using chi-square test.

(1) $$\text{spVL}=\beta 1*\text{variable}\;+\;\sum_{i=1}^{N}\beta_{i}*{\text{allele}}_{i}+\beta_{2}*\text{covariates}\;+\;ɛ1,$$

(2) $$\text{spVL}=\beta 1*\text{variable}\;+\;\sum_{i=1}^{N}\beta_{i}*{\text{allele}}_{i}+\beta_{2}*\text{covariates}\;+\;ɛ2.$$

All analyses were performed in R v3.5.1, and data were visualized using the ggplot2 v2.2.1 package (Wickham 2009).

## Supplementary Material

msz249_Supplementary_Data
