# Supplementary material for: HLA Heterozygote Advantage against HIV-1 Is Driven by Quantitative and Qualitative Differences in HLA Allele-Specific Peptide Presentation
Source: Mol Biol Evol. 2019 Oct 22;37(3):639–50. doi: 10.1093/molbev/msz249 (PMC7038656; doi:10.1093/molbev/msz249)
Supplement: msz249_Supplementary_Data [file msz249_supplementary_data.pdf]

Supplementary data for

HLA heterozygote advantage against HIV-1 is driven by quantitative and qualitative differences in HLA allele-specific peptide presentation

Jatin Arora [a], Federica Pierini [a], Paul J. McLaren [b, c], Mary Carrington [d, e], Jacques Fellay [f, g, h] & Tobias L. Lenz [a,*]

[a] Research Group for Evolutionary Immunogenomics, Max Planck Institute for Evolutionary Biology, 24306 Plön, Germany

[b] JC Wilt Infectious Diseases Research Center, National HIV and Retrovirology Laboratory, Public Health Agency of Canada, R3E 0W3, Winnipeg, Canada

[c] Department of Medical Microbiology and Infectious Diseases, University of Manitoba, R3E 0J9, Winnipeg Canada

[d] Basic Science Program, Frederick National Laboratory for Cancer Research, Frederick, MD 21702, USA.

[e] Ragon Institute of Massachusetts General Hospital, Massachusetts Institute of Technology and Harvard University, Cambridge, MA 02139-3583, USA.

[f] Global Health Institute, School of Life Sciences, École Polytechnique Fédérale de Lausanne, 1015 Lausanne, Switzerland

[g] Swiss Institute of Bioinformatics, 1015 Lausanne, Switzerland

[h] Precision Medicine Unit, Lausanne University Hospital and University of Lausanne, 1011 Lausanne, Switzerland

* Corresponding author. E-mail: lenz@post.harvard.edu

Supplementary data includes:

Figures S1-S12

Tables S1-S7

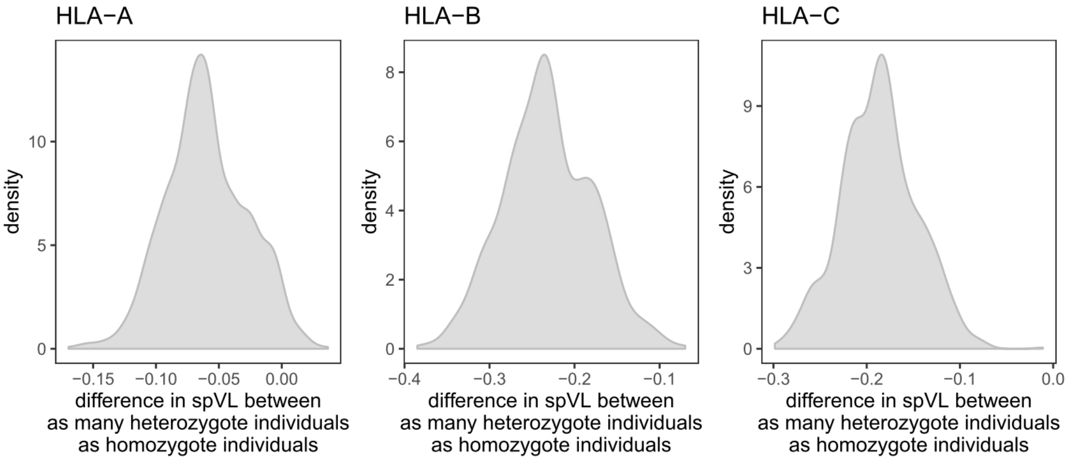

Fig. S1. Distribution of the difference in the viral load between HLA homozygous individuals and an equal number of randomly sampled HLA heterozygous individuals (1000 times). The viral load in HLA-B or HLA-C heterozygote individuals were always less compared to homozygote ones, while the same was not true for HLA-A.

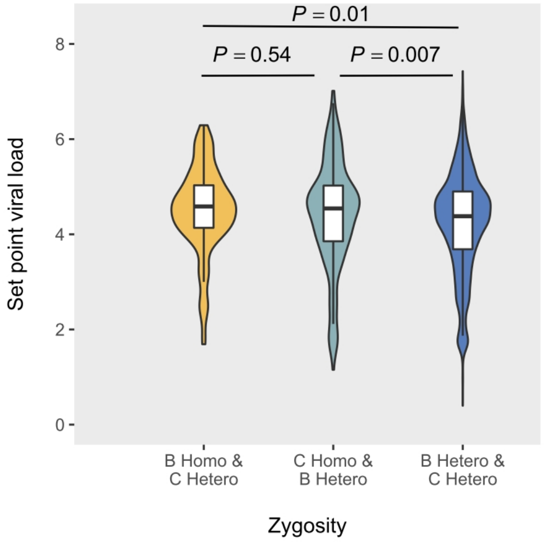

Fig. S2. Comparison of the set point viral load between individuals who are heterozygous for HLA-C only (N = 110), for HLA-B only (N = 295) and for both HLA-B and HLA-C (N = 5,368). *P* values from Wilcoxon rank sum test are shown.

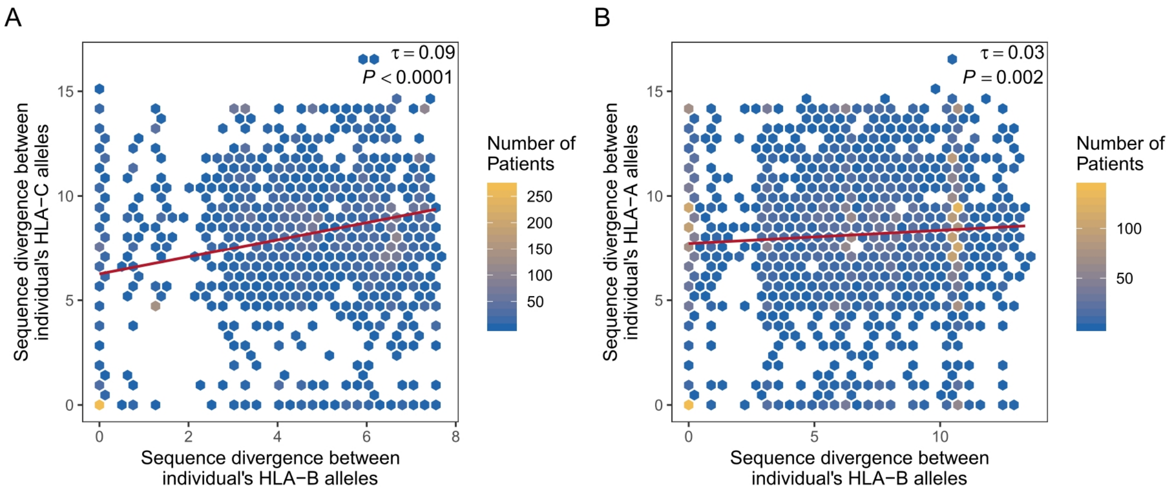

Fig. S3. Correlation between the sequence divergence of HLA-B alleles and the sequence divergence of HLA-C alleles (A), or HLA-A alleles (B), of an individual is shown. Individuals with similar parameter values are binned for better visualization. Kendall's estimate of correlation and Bonferroni-corrected *P*-value is shown.

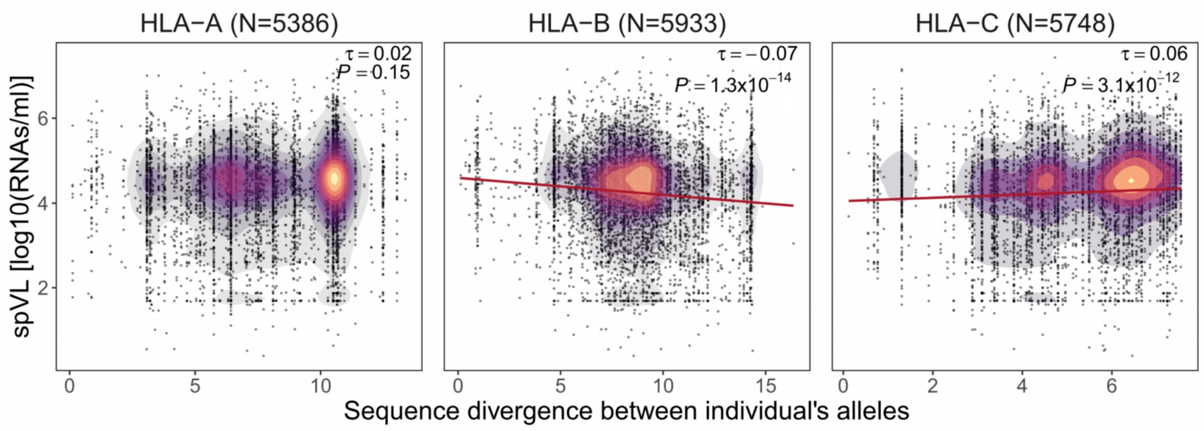

Fig. S4. Sequence divergence between individual's HLA alleles and viral load within HLA heterozygous individuals. Correlation between set point viral load (spVL) and sequence divergence between individual's HLA-A, HLA-B and HLA-C alleles is shown for heterozygous individuals. Color indicates the density of individuals. N indicates the number of heterozygous individuals. Kendall's estimate of correlation τ and Bonferroni-corrected *P*-value are shown.

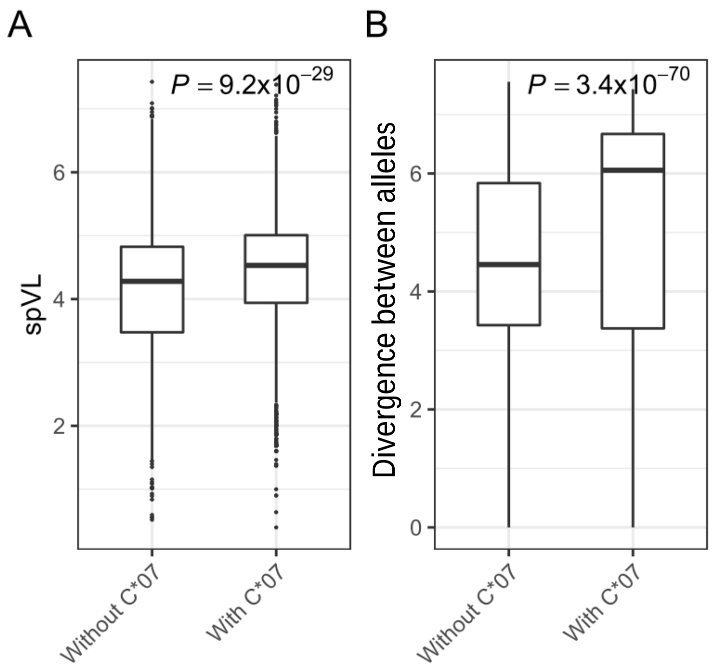

Fig. S5. Comparison of (A) set point viral load (spVL), and (B) HLA-C allele divergence (measured as Grantham distance between the two HLA-C alleles), between individuals who carried at least one allele belonging to the HLA-C*07 supertype (namely C*07:01, C*07:02 and C*07:04) and the ones who carried no alleles belonging to this supertype.

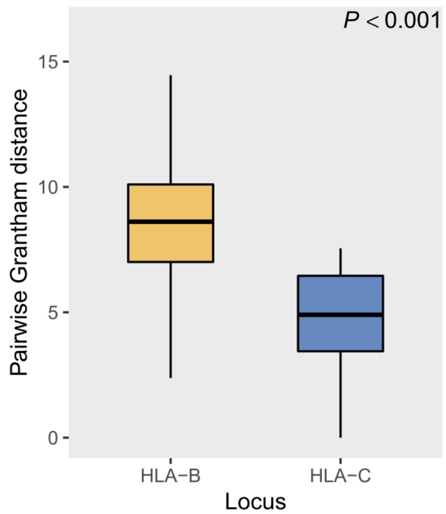

Fig. S6. Comparison of pairwise Grantham distance between individual's HLA-B and HLA-C alleles. *P*-value from Wilcoxon rank sum test are shown.

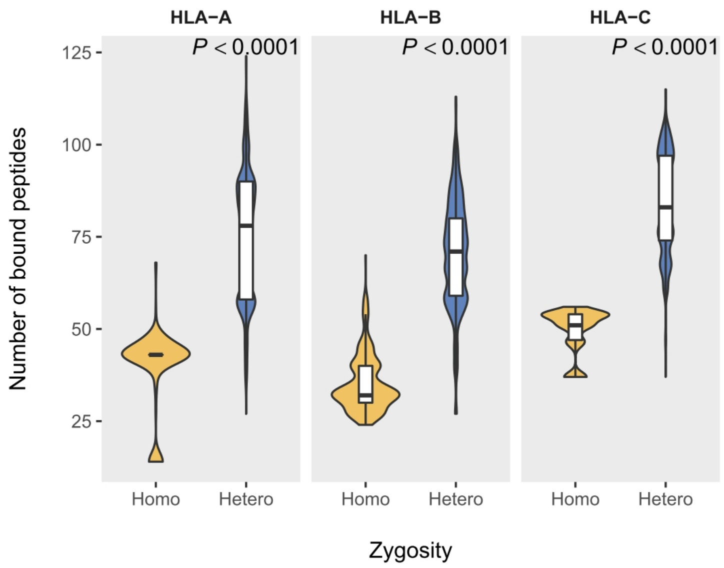

Fig. S7. HLA heterozygous individuals, whether for HLA-A, HLA-B or HLA-C, bound a significantly greater breadth of HIV-1 peptides with their corresponding alleles compared to homozygotes. *P*-value from Wilcoxon rank sum test are shown.

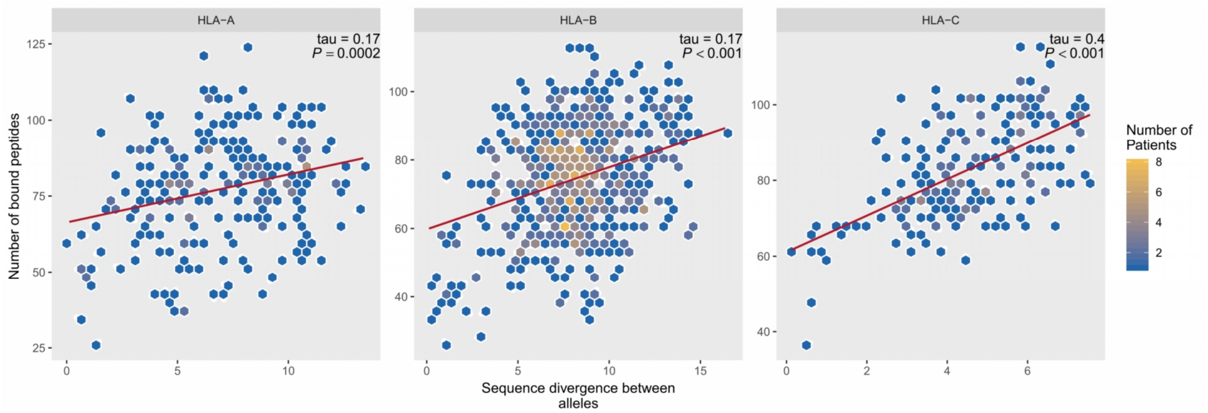

Fig. S8. Correlation between pairwise sequence divergence (Grantham distance) between an individual's HLA alleles and the breadth of bound HIV-1 peptides for HLA-A, HLA-B and HLA-C. Kendall's estimate of correlation τ and Bonferroni-corrected *P*-value are shown.

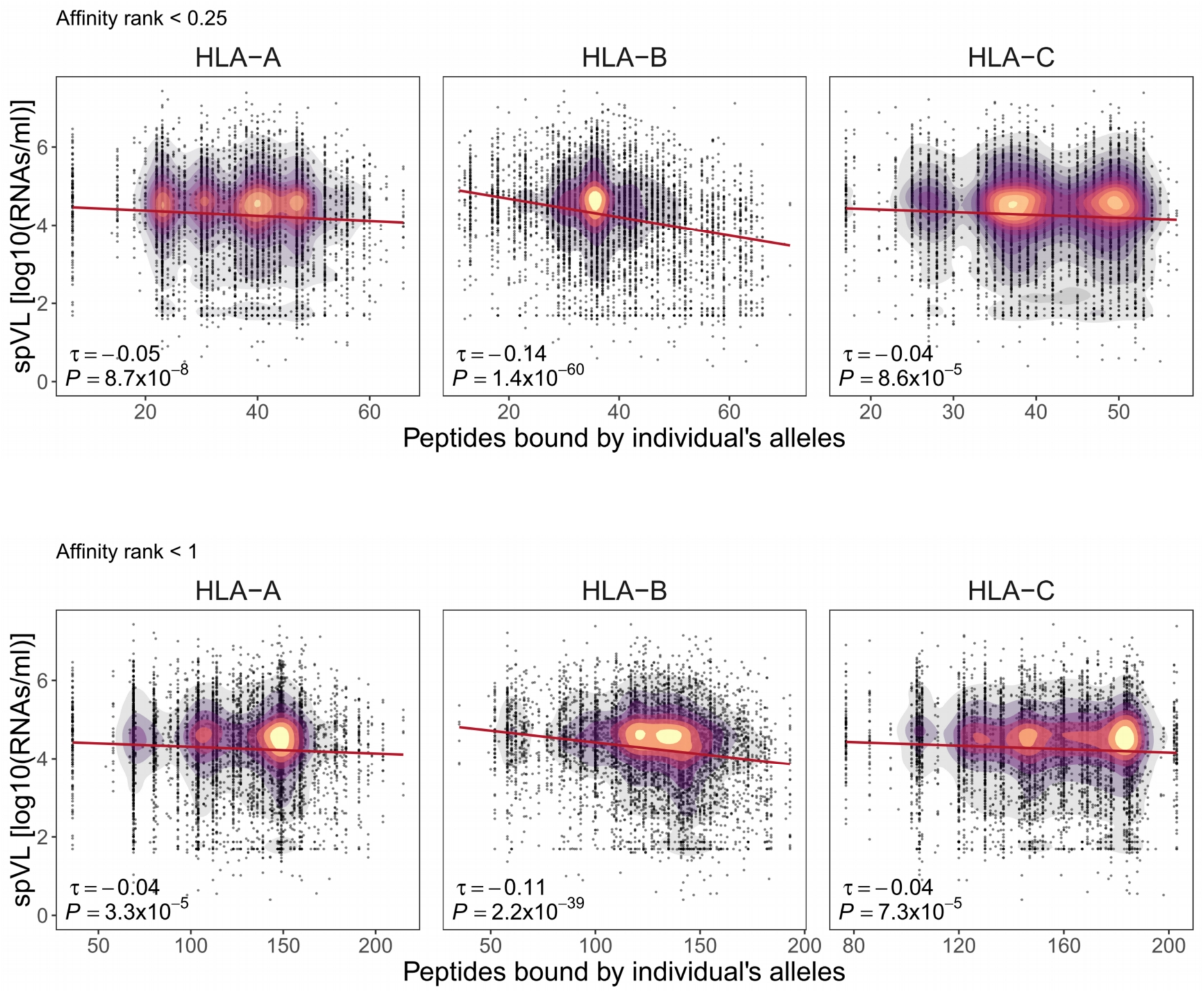

Fig. S9. Correlation between individual set point viral load (spVL; log10 HIV-1 RNA copies/ml of plasma) and the breadth of HIV-1 peptides predicted to be bound by HLA-A, HLA-B or HLA-C alleles, using a more stringent (rank < 0.25, top panel) and a more relaxed threshold (rank < 1, bottom panel) on the rank of predicted HLA-peptide affinity, are shown (including homo- and heterozygote individuals; N = 6,311).

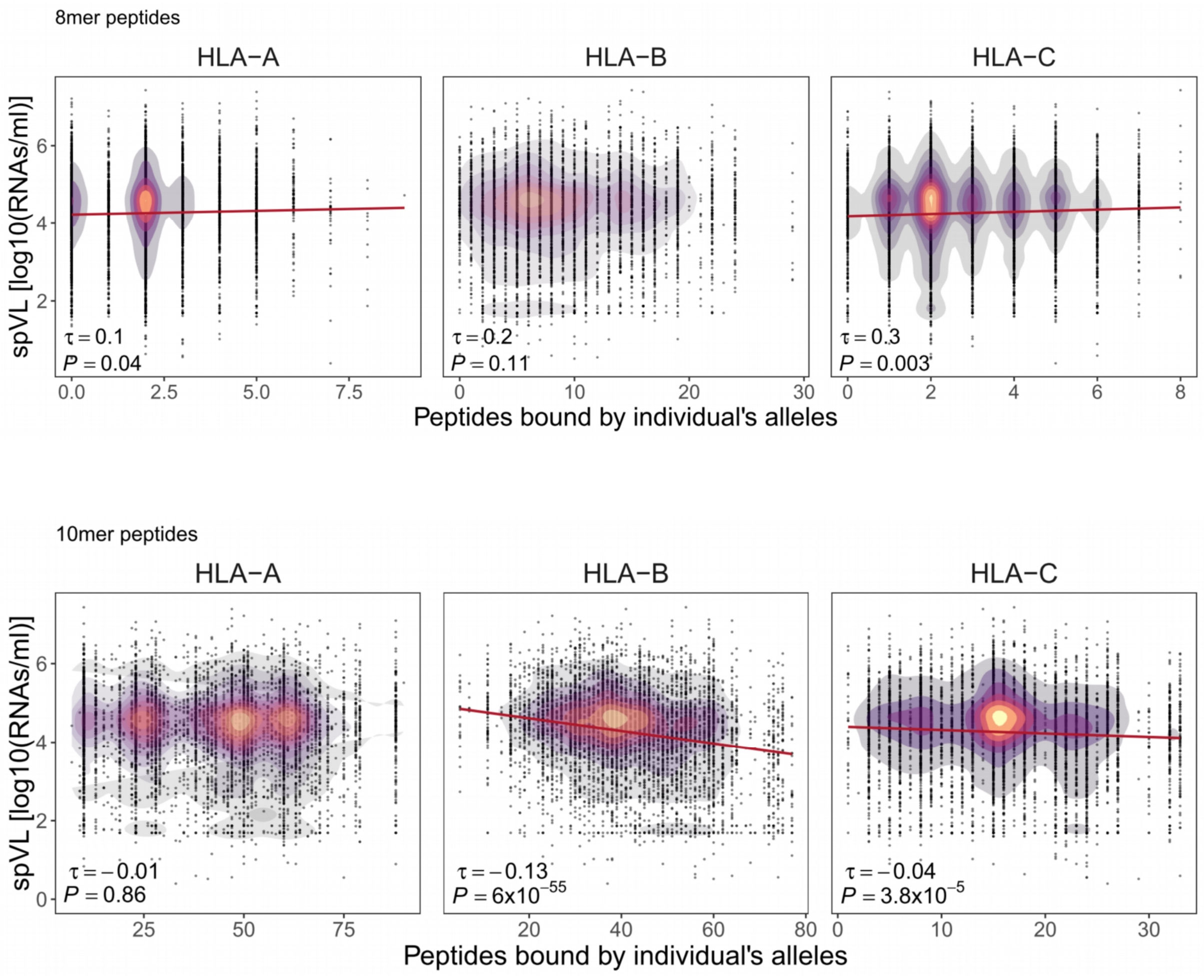

Fig. S10. Correlation between individual set point viral load (spVL; log10 HIV-1 RNA copies/ml of plasma) and the breadth of 8mer (top panel) and 10mer (bottom panel) HIV-1 peptides predicted to be bound by HLA-A, HLA-B and HLA-C alleles is shown (including homo- and heterozygote individuals; N = 6,311).

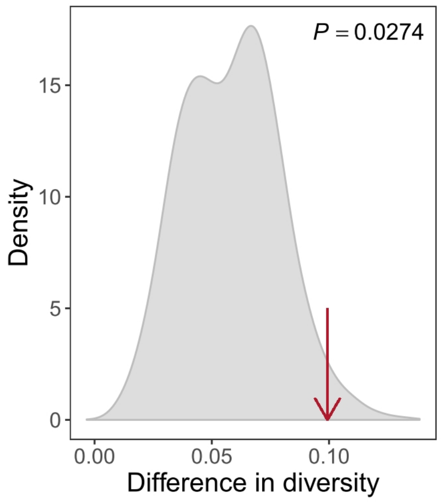

Fig. S11. The observed difference in the mean root-to-tip distance in groups of HLA-B heterozygous and homozygous individuals resided within the top 2.7% of distance distributions of 10,000 tree-pairs generated by permuting the individuals across zygosity groups. *P*-value is one-tailed.

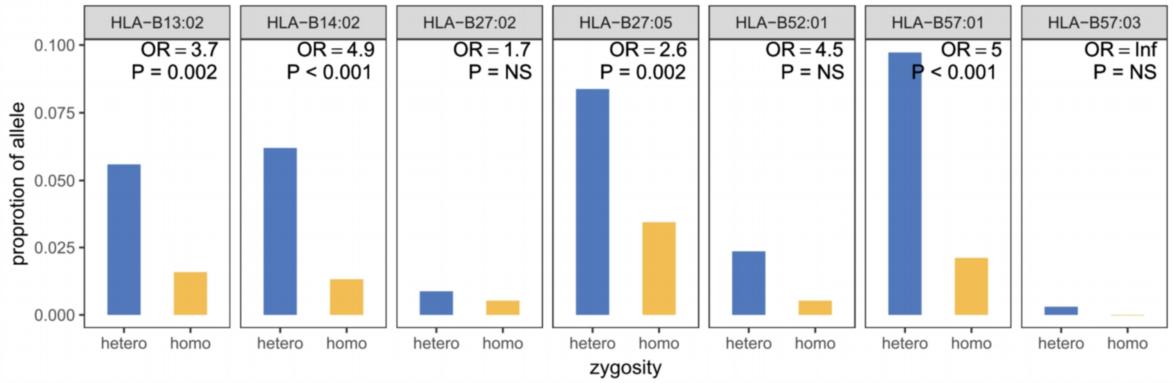

Fig. S12. Enrichment for protective HLA-B alleles in HLA-B heterozygous individuals compared to homozygous individuals. Odds ratio (OR) and *P*-value from Fisher exact test are shown.

Table S1. The HLA-B alleles represented in the dataset. There were 69 alleles at 4-digit resolution for the HLA-B gene represented in our dataset, which were predicted to bind a varying number of HIV-1 peptides (41 ± 10).

HLA allele	Number of bound HIV peptides
HLA-B07:02	32
HLA-B07:04	37
HLA-B07:05	34
HLA-B08:01	40
HLA-B13:01	65
HLA-B13:02	48
HLA-B14:01	45
HLA-B14:02	45
HLA-B15:01	45
HLA-B15:03	53
HLA-B15:05	48
HLA-B15:07	47
HLA-B15:08	38
HLA-B15:10	56
HLA-B15:16	49
HLA-B15:17	54
HLA-B15:18	51
HLA-B15:25	46
HLA-B15:27	49
HLA-B18:01	34
HLA-B27:02	28
HLA-B27:03	31
HLA-B27:04	31
HLA-B27:05	35
HLA-B27:07	27
HLA-B35:01	34
HLA-B35:02	41
HLA-B35:03	41
HLA-B35:08	33
HLA-B35:12	41
HLA-B35:17	33
HLA-B37:01	43
HLA-B38:01	58
HLA-B39:01	54
HLA-B39:06	38
HLA-B39:09	55
HLA-B39:10	42
HLA-B40:01	24
HLA-B40:02	26
HLA-B40:06	36
HLA-B41:01	44
HLA-B41:02	35
HLA-B42:01	42
HLA-B42:02	49
HLA-B44:02	27
HLA-B44:03	27

HLA-B44:04	25
HLA-B44:05	31
HLA-B45:01	43
HLA-B46:01	47
HLA-B47:01	49
HLA-B48:01	70
HLA-B49:01	35
HLA-B50:01	39
HLA-B51:01	30
HLA-B51:02	33
HLA-B51:05	35
HLA-B51:06	36
HLA-B51:08	36
HLA-B52:01	51
HLA-B53:01	48
HLA-B55:01	33
HLA-B56:01	39
HLA-B56:04	41
HLA-B57:01	54
HLA-B57:02	57
HLA-B57:03	56
HLA-B58:01	57
HLA-B73:01	33

Table S2. The HLA-A alleles represented in the dataset. There were 37 alleles at 4-digit resolution for the HLA-A gene represented in our dataset, which were predicted to bind a varying number of HIV-1 peptides (40 ± 11).

HLA allele	Number of bound HIV peptides
HLA-A01:01	14
HLA-A01:02	26
HLA-A01:03	15
HLA-A02:01	43
HLA-A02:02	40
HLA-A02:03	47
HLA-A02:05	47
HLA-A02:06	44
HLA-A02:11	51
HLA-A03:01	47
HLA-A03:02	41
HLA-A11:01	41
HLA-A11:02	41
HLA-A11:03	44
HLA-A23:01	39
HLA-A24:02	43
HLA-A24:07	47
HLA-A25:01	38
HLA-A26:01	28
HLA-A26:08	29
HLA-A29:01	35
HLA-A29:02	35
HLA-A30:01	62
HLA-A30:02	37
HLA-A30:04	45
HLA-A31:01	55
HLA-A32:01	68
HLA-A33:01	41
HLA-A33:03	46
HLA-A34:02	49
HLA-A36:01	21
HLA-A66:01	43
HLA-A68:01	34
HLA-A68:02	38
HLA-A69:01	41
HLA-A74:01	52
HLA-A80:01	23

Table S3. The HLA-C alleles represented in the dataset. There were 27 alleles at 4-digit resolution for the HLA-C gene represented in our dataset, which were predicted to bind a varying number of HIV-1 peptides (50 ± 5).

HLA allele	Number of bound peptides
HLA-C01:02	51
HLA-C02:02	56
HLA-C02:06	55
HLA-C03:02	52
HLA-C03:03	47
HLA-C03:04	47
HLA-C04:01	37
HLA-C04:03	48
HLA-C04:07	37
HLA-C05:01	46
HLA-C06:02	54
HLA-C07:01	54
HLA-C07:02	51
HLA-C07:04	51
HLA-C08:01	52
HLA-C08:02	54
HLA-C12:02	48
HLA-C12:03	54
HLA-C14:02	50
HLA-C15:02	55
HLA-C15:04	53
HLA-C15:05	51
HLA-C16:01	53
HLA-C16:02	53
HLA-C16:04	47
HLA-C17:01	65
HLA-C18:01	43

Table S4. We constructed a linear model that included the heterozygosity at HLA-A, HLA-B and HLA-C as 3 predictor variables and viral load as dependent variable. The significance of the β-coefficient for individual HLA genes supports independent protective effects of heterozygosity at HLA-B and HLA-C, but not HLA-A on disease progression.

Effect of HLA heterozygosity on spVL		
Locus	β-coefficient	P-value
HLA-A	-0.04	0.31
HLA-B	-0.15	0.02
HLA-C	-0.13	0.01

Table S5. We constructed a linear model that included Grantham distance between individual's HLA-A, HLA-B and HLA-C allele pairs as 3 predictor variables and the viral load as dependent variable. The β-coefficient of HLA genes shows the strength of the effect of Grantham distance between alleles of an HLA gene and the viral load in an individual.

Sequence divergence between HLA alleles		
Locus	β-coefficient	P-value
HLA-A	0.001	0.64
HLA-B	-0.04	1.6×10^{-22}
HLA-C	0.018	0.003

Table S6. We constructed a linear model that included the number of HIV-1 peptides bound by individual's HLA-A, HLA-B and HLA-C molecule variants as 3 predictor variables and the viral load as dependent variable. The β-coefficient of HLA genes shows the strength of the effect of the number of HLA-bound peptides on the viral load in an individual.

Number of HLA-bound peptides		
Locus	β-coefficient	P-value
HLA-A	-0.003	7.8×10^{-6}
HLA-B	-0.012	1.3×10^{-45}
HLA-C	-0.002	0.01

Table S7. Summary of the represented sample cohorts.

GWAS study	#Samples	Origin
Fellay J, et al. (2009)	1304	Europe / Australia
Migueles SA, et al. (2000), Dean M, et al. (1996)	1034	USA
Limou S, et al. (2009), Le Clerc S, et al. (2009)	581	France
Migueles SA, et al. (2000), Dean M, et al. (1996)	729	USA
Pereyra F, et al. (2010)	576	USA
Pereyra F, et al. (2010)	576	USA
Pereyra F, et al. (2010)	503	USA
Migueles SA, et al. (2000), Dean M, et al. (1996)	364	USA
Van Manen D, et al. (2011)	383	Netherlands
Urban Health Study: Genetics Cohort (UHSGC)	261	USA

The dataset includes a total of 6,311 samples. Shown are the GWAS study cohorts, the number of the samples that contribute to this study, and their origin. For more detailed information see McLaren et al. 2015.
